# Obesity associated alterations in the biology of adipose stem cells mediate enhanced tumorigenesis by estrogen dependent pathways

**DOI:** 10.1186/bcr3569

**Published:** 2013-10-31

**Authors:** Amy L Strong, Thomas A Strong, Lyndsay V Rhodes, Julie A Semon, Xiujuan Zhang, Zhenzhen Shi, Shijia Zhang, Jeffrey M Gimble, Matthew E Burow, Bruce A Bunnell

**Affiliations:** 1 Center for Stem Cell Research and Regenerative Medicine, Tulane University School of Medicine, 1430 Tulane Avenue, SL-99, New Orleans, LA 70112, USA; 2 Department of Medicine, Section of Hematology and Medical Oncology, Tulane University Health Sciences Center, New Orleans, LA 70112, USA; 3 Stem Cell Biology Laboratory, Pennington Biomedical Research Center, Louisiana State University System, Baton Rouge, LA 70808, USA; 4 Department of Pharmacology, Tulane University School of Medicine, New Orleans, LA 70112, USA

## Abstract

**Introduction:**

Obesity has been associated with increased incidence and mortality of breast cancer. While the precise correlation between obesity and breast cancer remains to be determined, recent studies suggest that adipose tissue and adipose stem cells (ASCs) influence breast cancer tumorigenesis and tumor progression.

**Methods:**

Breast cancer cells lines were co-cultured with ASCs (n = 24), categorized based on tissue site of origin and body mass index (BMI), and assessed for enhanced proliferation, alterations in gene expression profile with PCR arrays, and enhanced tumorigenesis in immunocompromised mice. The gene expression profile of ASCs was assess with PCR arrays and qRT-PCR and confirmed with Western blot analysis. Inhibitory studies were conducted by delivering estrogen antagonist ICI182,780, leptin neutralizing antibody, or aromatase inhibitor letrozole and assessing breast cancer cell proliferation. To assess the role of leptin in human breast cancers, Oncomine and Kaplan Meier plot analyses were conducted.

**Results:**

ASCs derived from the abdominal subcutaneous adipose tissue of obese subjects (BMI > 30) enhanced breast cancer cell proliferation *in vitro* and tumorigenicity *in vivo*. These findings were correlated with changes in the gene expression profile of breast cancer cells after co-culturing with ASCs, particularly in estrogen receptor-alpha (ESR1) and progesterone receptor (PGR) expression. Analysis of the gene expression profile of the four groups of ASCs revealed obesity induced alterations in several key genes, including leptin (LEP). Blocking estrogen signaling with ICI182,780, leptin neutralizing antibody, or letrozole diminished the impact of ASCs derived from obese subjects. Women diagnosed with estrogen receptor/progesterone receptor positive (ER^+^/PR^+^) breast cancers that also expressed high levels of leptin had poorer prognosis than women with low leptin expression.

**Conclusion:**

ASCs isolated from the abdomen of obese subjects demonstrated increased expression of leptin, through estrogen stimulation, which increased breast cancer cell proliferation. The results from this study demonstrate that abdominal obesity induces significant changes in the biological properties of ASCs and that these alterations enhance ER^+^/PR^+^ breast cancer tumorigenesis through estrogen dependent pathways.

## Introduction

Breast cancer is the most frequently diagnosed cancer in women and the second leading cause of cancer deaths in the United States. While many risk factors increase the incidence of breast cancer, obesity is among one of the most important risk factors for breast cancer in postmenopausal women as it not only increases the incidence of breast cancer but also the mortality rate due to poor prognosis and outcome [1-4]. Obesity has been shown to affect prognosis through multiple mechanisms, including enhanced metastasis rate and drug resistance [5,6]. Furthermore, recent studies have demonstrated a stronger correlation between abdominal obesity and breast cancer [7,8].

While the precise link between obesity and breast cancer remains to be determined, previous studies have described the activation of adipose stem cells (ASCs) in the presence of breast cancer to contribute to its pathogenesis [9,10]. ASCs are mesenchymal lineage stem cells that are recruited to the tumor or sites of inflammation and are essential components that establish the tumor microenvironment [11-13]. This recruitment enhances tumor growth through the secretion of an abundance of growth factors from ASCs, such as IL-6, CCL5 and PDGR, which have been shown to contribute to both the breast cancer tumorigenesis and the metastasis of breast cancer cells [14,15].

While previous studies have determined that ASCs play an integral role in the progression of breast tumors, the impact of obesity and abdominal obesity on the relationship between cancer and ASCs has not been investigated. Studies have revealed that ASCs isolated from subjects with vastly different body mass indices display differences in their secretory profile, angiogenic potential and invasive capacity [16,17]. Recent work by Kolonin *et al*. demonstrated increased numbers of ASC in obese mice relative to lean mice, and this increase in ASC number enhances vascularization and proliferation of malignant cells [13]. The results from these studies suggest that the local microenvironment from which these ASCs are isolated can influence their gene expression profiles. In addition, the site of origin of the adipose tissue from which the ASCs are derived may alter essential cellular signaling pathways that may directly influence breast cancer tumorigenesis.

This study investigated the impact of ASCs, isolated from different subcutaneous adipose depots in subjects with increasing body mass index (BMI), on the growth, gene expression profile and tumorigenesis of breast cancer cells. The results from this study demonstrated that ASCs isolated from abdominal depots of obese subjects (BMI >30) enhanced breast cancer cell growth and tumorigenesis via an estrogen mediated response.

## Methods

### Human subjects

All protocols were reviewed and approved by the Pennington Biomedical Research Center Institutional Review Board and all human participants provided written informed consent (PBRC #23040). Human ASCs were obtained from 24 Caucasian females (4 groups, 6 donors per group) undergoing elective liposuction procedures, as previously described [18,19]. Briefly, ASCs were isolated from processed lipoaspirates from subcutaneous abdominal adipose tissue of obese (Ob^+^Ab^+^) or non-obese (Ob^-^Ab^+^) subjects and from non-abdominal subcutaneous adipose depots of obese (Ob^+^Ab^-^) and non-obese (Ob^-^Ab^-^) subjects. Liposuction aspirates were incubated in 0.1% type I collagenase (Sigma) and 1% powered bovine serum albumin (BSA, fraction V; Sigma, St. Louis, MO, USA) dissolved in 100 ml of phosphate buffered saline (PBS) supplemented with 2 mM calcium chloride. This mixture was placed in a 37°C shaking water bath at 75 rpm for 60 minutes and then centrifuged to remove oil, fat, primary adipocytes and collagenase solution, leaving behind a pellet of cells. Cells were resuspended in complete culture media (CCM), which consisted of α-Minimal Essential Medium (αMEM; GIBCO; Grand Island, NY, USA), 20% fetal bovine serum (Atlanta Biologicals, Lawrenceville, GA, USA), 100 units per ml penicillin/100 μg/mL streptomycin (P/S; GIBCO), and 2 mM L-glutamine (GIBCO), and plated on 150 cm^2^ culture dishes. Fresh CCM was added every two to three days until cells achieved 80 to 90% confluence, at which time cells were harvested with 0.25% trypsin/1 mM EDTA (GIBCO) and cryopreserved prior to experimental use. Non-abdominal subcutaneous adipose tissue was isolated from the hip, knee, thigh, ankle, flank, upper toroso, scapula, forearm, arm and back. The mean BMI for each of the four donor groups was as follows: Ob^+^Ab^+^ (32.7 ± 3.7), Ob^+^Ab^-^ (31.1 ± 0.6), Ob^-^Ab^+^ (22.7 ± 1.9) and Ob^-^Ab^-^ (22.5 ± 1.2). The mean age of the subjects for each group of donors was as follows: Ob^+^Ab^+^ (42.5 ± 8.9), Ob^+^Ab^-^ (44.0 ± 12.4), Ob^-^Ab^+^ (38.8 ± 7.0) and Ob^-^Ab^-^ (52.4 ± 18.0). No statistical significance in age was observed between the donor groups.

### Cell culture

#### ASCs

Frozen vials of ASCs were thawed and cultured on 150 cm^2^ culture dishes (Nunc, Rochester, NY, USA) in 25 ml CCM and incubated at 37°C with 5% humidified CO_2_. After 24 hours, viable cells were harvested with 0.25% trypsin/1 mM EDTA and replated at 100 cells/cm^2^ in CCM. Media was changed every two to three days. For all experiments, sub-confluent cells (≤70% confluent) between passages 2 to 6 were used.

To characterize the cells, ASCs were induced to undergo osteogenic and adipogenic differentiation. For osteogenic differentiation, ASCs were cultured in six-well plates in CCM until 70% confluent and media was replaced with fresh media containing osteogenic supplements, consisting of 50 μM ascorbate 2-phosphate (Sigma), 10 mM β-glycerol phosphate (Sigma) and 10 nM dexamethasone. After three weeks, cells were fixed in 10% formalin for 1 hour at 4°C and stained for 10 minutes with 40 mM Alizarin Red (pH 4.1) to visualize calcium deposition in the extracellular matrix. Images were acquired at 4× magnification on Nikon Eclipse TE200 (Melville, NY, USA) with Nikon Digital Camera DXM1200F using the Nikon ACT-1 software version 2.7. For adiogenic differentiation, ASCs were cultured in six-well plates in CCM until 70% confluent, and media was replaced with fresh media containing adipogenic supplements, consisting of 0.5 μM dexamethasone (Sigma), 0.5 mM isobuytlmethylxanthine (Sigma) and 50 μM indomethacin (Sigma). After three weeks, cells were fixed in 10% formalin for 1 hour at 4°C, stained for 10 to 15 minutes at room temperature with Oil Red O (Sigma) to detect neutral lipid vacuoles, and images were acquired at 10× magnification on Nikon Eclipse TE200 with Nikon Digital Camera DXM1200F using Nikon ACT-1 software version 2.7.

To determine the ability to form colony forming units (CFU), ASCs at passage 3 were plated at a density of 100 cells on a 10 cm^2^ plate in CCM and incubated for 14 days. Plates were rinsed three times with PBS, and 10 mL of 3% crystal violet (Sigma) was added for 30 minutes at room temperature. Plates were washed three times with PBS and once with tap water. Each experiment was performed in triplicate.

Analysis by flow cytometry of the cell surface marker profile was conducted by harvesting ASCs with 0.25% trypsin/1 mM EDTA for three to four minutes at 37°C. A total of 3×10^5^ cells were concentrated by centrifugation at 500 *x* g for five minutes, suspended in 50 μl PBS and labeled with the primary antibodies. The following primary antibodies were used: Anti-CD45-PeCy7, anti-CD11b-PeCy5, anti-CD166-PE, anti-CD105-PE, anti-CD90-PeCy5, anti-CD34-PE, isotype-control FITC human IgG1 and isotype-control PE human IgG2a were purchased from Beckman Coulter (Indianapolis, IN, USA). Anti-CD44-APC was purchased from BD Biosciences (San Jose, CA, USA). The samples were incubated for 30 minutes at room temperature and washed three times with PBS. The samples were then analyzed with Galios Flow Cytometer (Beckman Coulter, Brea, CA, USA) running Kaluza software (Beckman Coulter). To assay cells by forward and side scatter of light, FACScan was standardized with microbeads (Dynosphere Uniform Microspheres; Bangs Laboratories Inc.; Thermo Scientific, Waltham, MA, USA). At least 10,000 events were analyzed and compared with isotype controls.

#### Breast cancer cell lines

MCF7 and MDA-MB-231 cells were obtained from the American Type Culture Collection (Manassas, VA, USA) and cultured in Dulbecco’s Modified Eagle’s Medium (DMEM; GIBCO), supplemented with 10% FBS and P/S. Cells were grown at 37°C with 5% humidified CO_2_, fed every two to three days, and split 1:4 to 1:6 when they reached 90% confluency.

### Synthesis of GFP breast cancer cells

To produce retroviruses carrying green fluorescent protein (GFP) and neomycin resistance (neo), 293T cells were transfected by means of a modified calcium chloride transfection protocol when cells reached 90 to 95% confluency. The following amount of DNA was used to transfect cells on a 10 cm plate: 10 μg pMSCVneo-GFP vector, 10 μg pVPACK-Gp-dl packaging plasmid, and 10 μg pCI-VSV-G envelope-encoding plasmid. Twenty-four hours after transfection, cells were washed with PBS, replaced with fresh media, and collected after 48 hours. To transduce MCF7 or MDA-MB-231 cells, conditioned media containing retrovirus was added to MCF7 or MDA-MB-231 cells at 70% confluency. MCF7 cells were selected with 1 mg/ml of Genticin (Invitrogen, Carlsbad, CA, USA), while MDA-MB-231 cells were selected with 500 μg/ml of Genticin for two week and GFP expression was verified with flow cytometry.

### Breast cancer cell and ASC co-culture

**Figure 1:**
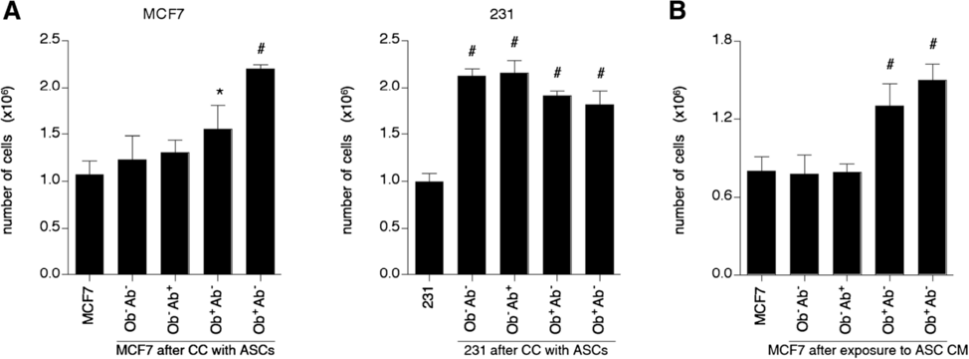
**Direct co-culture of breast cancer cells with ASCs result in increased proliferation *in vitro*. (A)** After seven days of co-culture (CC) with six adipose stem cells (ASC) donors per group, quantification of MCF7 cells and MDA-MB-231 (231) cells was based on the percentage of GFP^+^ cells in the population multiplied by the total number of cells in each condition. **(B)** To determine the influence of ASCs on MCF7 cells indirectly, conditioned media (CM) from pooled ASCs (n = 6 donors per group) were collected and added to MCF7 cells. After seven days, the total number of MCF7 cells was counted. Values reported are the mean of three independent experiments, each performed in triplicate. Bars, ± SD. *, *P* <0.05; ^#^, *P* <0.01.

MCF7 cells or MDA-MB-231 cells were co-cultured with Ob^-^Ab^-^ (n = 6), Ob^-^Ab^+^ (n = 6), Ob^+^Ab^-^(n = 6), or Ob^+^Ab^+^ (n = 6) ASCs (1:1 ratio) at 200 cells/cm^2^ in DMEM supplemented with 10% FBS and P/S. After seven days, cells were harvested, washed and analyzed by flow cytometry. The percentage of GFP positive cells (MCF7 cells or MDA-MB-231) was determined with Gallios Flow Cytometer running Kaluza software (Beckman Coulter, Brea, CA, USA) (Figure 1A). Where indicated, MCF7 cells were co-cultured with Ob^-^Ab^-^, Ob^-^Ab^+^, Ob^+^Ab^-^, or Ob^+^Ab^+^ ASCs (1:1 ratio) grown in CCM made with charcoal dextran stripped-FBS (CDS-FBS) with or without supplemental estrogen (E_2_; 10 nM), leptin neutralizing antibody (R&D Systems; Minneapolis, MN, USA), or letrozole (Sigma). For RNA isolation, MCF7 cells were sorted after co-culture with the Becton-Dickinson FACSVantage SE Cell Sorter with DiVa option (BD Biosciences, Franklin Lakes, NJ, USA) and analyzed with the DiVa software v5.02 (BD).

### ASC conditioned media

ASCs, pooled from six donors per group, were plated on at 150 cm^2^ culture dish at 100 cells/cm^2^. After overnight culture, media was replaced with serum free αMEM medium. After seven days, conditioned media was collected and filtered to remove debris. ASC conditioned media from each group was plated on top of MCF7 cells set up in triplicates. After seven days, the total number of MCF7 cells were counted with a hemocytometer. Three independent experiments were conducted, each in triplicate.

### RT^2^ profiler™ PCR arrays

#### Breast cancer PCR arrays

Total cellular RNA was extracted from FACS purified MCF7 cells after co-culture with a pool of ASCs (n = 6 per group) or MCF7 control cells (not co-cultured) using RNeasy Mini Kit (Qiagen, Valencia, CA, USA) and treated with DNase I digestion (Qiagen) according to manufacturer’s instructions. One μg of RNA was converted to cDNA with the RT^2^ First Strand Kit (SABiosciences, Frederick, MD, USA) according to the manufacturer’s protocol. Gene expression profiling was performed using the Breast Cancer RT^2^ Profiler PCR Array (SABiosciences) and RT^2^ qPCR Master Mix (SABiosciences). PCR amplification was performed in a Bio-Rad CFX96 Real-Time System (Bio-Rad, Hercules, CA, USA). The reaction conditions were as follows: 95°C for 10 minutes, 40 cycles of 95°C for 15 sec and 60°C for 1 minute, followed by a dissociation curve. At the completion of the reaction, C_t_ values were determined, and ΔΔ C_t_ and fold change were determined using the RT^2^ Profiler PCR Array Data Analysis web portal (SABiosciences). Genes whose mRNA levels increased or decreased more than two-fold in MCF7 cells after co-culture with ASCs relative to MCF7 cells without co-culture were considered differentially expressed (*P* <0.05).

#### Obesity PCR arrays

Ob^-^Ab^-^ (n = 6), Ob^-^Ab^+^ (n = 6), Ob^+^Ab^-^ (n = 6) or Ob^+^Ab^+^ (n = 6) ASCs were expanded in CCM and collected for RNA extraction using the RNeasy Mini Kit, and the total cellular RNA was treated with DNase I per the manufacturer’s instructions. One μg of RNA was converted to cDNA with the RT^2^ First Strand Kit according to the manufacturer’s protocol. Gene expression profiling was performed using the Obesity RT^2^ Profiler PCR Array (SABiosciences) and RT^2^ qPCR Master Mix. Reaction settings and analysis was conducted as described above. Genes whose mRNA levels increased or decreased more than two-fold (*P* <0.05) in MCF7 cells after co-culture with ASCs relative to MCF7 cells without co-culture were considered differentially expressed.

### Western blot

MCF7 cells and FACS sorted MCF7 cells after co-cultured with ASC donors (n = 6 per group) were incubated in phosphatase and protease inhibitors (Pierce, Thermo Scientific, Rockford, IL, USA), lysed with RIPA buffer (Pierce), and centrifuged. Cell lysate was also obtained from Ob^-^Ab^-^ (n = 6), Ob^-^Ab^+^ (n = 6), Ob^+^Ab^-^ (n = 6), or Ob^+^Ab^+^ (n = 6) ASCs cultured in CCM made with charcoal dextrose stripped FBS (Atlanta Biologicals, Lawrenceville, GA, USA). Where indicated, ASCs were treated with 10 nM 17β-estradiol (E_2_, Sigma, St. Louis, MO, USA) and/or 100 nM ICI182,780 (Sigma), and cell lysate was obtained. A total of 20 μg of protein was fractionated on 4 to 12% SDS-polyacrylamide gels (Invitrogen) and transferred to nitrocellulose membranes (Invitrogen). The blots were blocked with blØk Noise Canceling Reagents (Millipore, Billerica, MA, USA) and probed using primary antibodies incubated overnight at 4°C, washed with phosphate-buffered solution with 0.01% TWEEN-20 (PBST), followed by a secondary antibody conjugated to horseradish peroxidase (HRP), washed with PBST and visualized with chemiluminescence reagent (Invitrogen) on an ImageQuant LAS 4000 (GE Healthcare Life Science; Piscataway, NJ, USA).

Antibodies against cyclin dependent kinase inhibitor 2A (CDKN2A), estrogen receptor-alpha (ESR1), and progesterone receptor (PGR) were obtained from Santa Cruz Biotechnology (Santa Cruz, CA, USA). Anti-secreted frizzled-related protein 1 (anti-SFRP1), anti-mouse-HRP, anti-rabbit-HRP antibodies were purchased from Abcam (Cambrige, MA). Anti-glutathione S-transferase P (anti-GSTP1) was purchased from Cell Signaling Technologies (Danvers, MA, USA), anti-actin was purchased from Sigma, and anti-leptin was purchased from R&D Systems.

### In vivo tumorigenicity assay

All procedures involving animals were conducted in compliance with State and Federal law, standards of the US Department of Health and Human Services, and guidelines established by Tulane University Institutional Animal Care and Use Committee (IACUC). All protocols were approved by the Tulane IACUC.

SCID/beige (CB17.Cg-Prkdc^scid^Lyst^bg-J^/Crl) immunocompromised female ovariectomized mice (five weeks old) were obtained from Charles River Laboratories (Wilmington, MA, USA). Mice were divided into treatment groups of five animals, with or without estrogen: MCF7 only, MCF7 plus Ob^-^Ab^-^ ASCs (n = 6 donors), MCF7 plus Ob^-^Ab^+^ ASCs (n = 6 donors), MCF7 plus Ob^+^Ab^-^ ASCs (n = 6 donors), and MCF7 plus Ob^+^Ab^+^ ASCs (n = 6 donors). Where indicated, estradiol pellets (0.72 mg, 60-day release, Innovative Research of America, Sarasota, FL, USA) were implanted subcutaneously in the lateral area of the neck.

MCF7 cells (10^6^) alone or MCF7 cells (10^6^) in combination with ASCs (10^6^) suspended in a total volume of 50 μl of sterile PBS were mixed with 100 μl of reduced growth factor Matrigel (BD Biosciences, Bedford, MA, USA). Cells were injected subcutaneously into the fifth mammary fat pad on both sides. All procedures in animals were carried out under anesthesia using a mixture of isoflurane and oxygen delivered by mask.

Tumor size was measured every three days using digital calipers and calculated as previously described [20]. At necropsy, animals were euthanized by cervical dislocation after exposure to CO_2_. Tumors were removed and frozen in liquid nitrogen or fixed in 10% neutral buffered formalin and paraffin embedded for further analysis.

### Immunohistochemistry

Formalin-fixed, paraffin-embedded (FFPE) tumor sections were deparaffinized, rehydrated in a graded solution of Sub-X solutions, stained with hematoxylin and eosin or quenched with 0.3% H_2_O_2_ (Sigma), rinsed with PBST, blocked with 1% BSA and stained with primary antibodies against Ki-67 (Abcam) or human progesterone receptor (PGR; DAKO North America, Inc., Carpinteria, CA, USA) overnight at 4°C. Each tumor section was subsequently washed in PBST, incubated with appropriate HRP-conjugated secondary antibody for one hour at room temperature, and washed with PBST. For colorimetric staining, slides were then incubated in 3,3'-Diaminobenzidine (DAB; Vector Laboratories; Burlingame, CA, USA), washed with PBST, counterstained with hematoxylin, and rinsed with deionized water. Slides were sealed with Permount Mounting Medium (Sigma). For apoptosis analysis, the TACS-XL *in situ* Apopotosis Detection Kit (R&D Systems) was used according to the manufacturer’s instructions. After staining, tumor sections were counterstained and sealed as mentioned above. Images were acquired at 10× and 40×. Quantification of the percentage of positivity was assessed using ImageScope (Aperio, Vista, CA, USA) and determined by the percentage of positive pixels divided by the total number of pixels in a given section.

### Quantitative Reverse Transcription-Polymerase Chain Reaction (qRT-PCR)

Ob^-^Ab^-^ (n = 6 donors), Ob^-^Ab^+^ (n = 6 donors), Ob^+^Ab^-^ (n = 6 donors), or Ob^+^Ab^+^ (n = 6 donors) ASCs cultured in CCM were collected for total cellular RNA extraction using a RNeasy Mini Kit. Where indicated, ASCs were cultured in CCM containing charcoal dextrose-stripped FBS, with or without supplementation with 10 nM E_2_ and/or 100 nM ICI182,780. RNA was then purified with DNase I digestion (Invitrogen), and reverse transcribed using the SuperScript VILO cDNA synthesis kit (Invitrogen). Quantitative real-time PCR was performed using the EXPRESS SYBR GreenER qPCR SuperMix Kit (Invitrogen) according to the manufacturer’s instructions. The following primer set sequence for leptin (forward 5′-gaagaccacatccacacacg-3′, reverse 5′-agctcagccagacccatcta-3′) and aromatase (forward 5′-cagaggccaagagtttgagg-3′, reverse 5′-acactagcaggtccctttgg-3′) were used. β-actin (forward 5′-caccttctacaatgagctgc-3′ and reverse 3′-tcttctcgatgctcgacgga-5′) was used as an internal reference point. At the completion of the reaction, ΔΔC_t_ was calculated to quantify mRNA expression.

### Oncomine analysis

A set of 440 normal breast tissues and invasive ductal carcinomas (IDC) deposited by The Cancer Genome Atlas (TCGA) was analyzed using the Oncomine Research Edition to assess leptin expression. Details of the standardized normalization techniques and statistical calculations can be found on the Oncomine website (http://www.oncomine.com).

### KM plot analysis

To determine the five-year relapse-free survival of patients diagnosed with breast cancer based on leptin expression, an online survival analysis tool was utilized and can be found on the Kaplan-Meier Plotter website (http://www.kmplot.com). Details of the standardized normalization techniques and characterization of high or low expression have been previously described [21].

### Statistical analysis

All values are presented as means ± standard deviation (SD). The statistical differences among two or more groups were determined by ANOVA, followed by *post-hoc* Dunnet multiple comparison tests versus the respective control group. The statistical differences between two groups were performed by Student’s *t*-test. Statistical significant was set at *P* <0.05. Analysis was performed using Prism (Graphpad Software, San Diego, CA, USA).

## Results

### Characterization of ASCs

ASCs were isolated from lipoaspirates of obese (Ob^+^) and non-obese (Ob^-^) subjects undergoing elective plastic surgery and from the subcutaneous abdominal adipose tissue (Ab^+^) or non-abdominal subcutaneous depots (Ab^-^). Each ASC donor (n = 24), irrespective of the ASC group the donor was categorized into, was analyzed for the expression of cell surface markers and were positive for CD44, CD90, CD105 and CD166 and negative for CD34, CD45 and CD11b determined with flow cytometry (Additional file 1). Each group of ASCs was able to generate colony-forming units and undergo osteogenesis and adipogenesis (Additional file 1). No differences were observed among the four groups for ASC differentiation or self-renewal capacity as defined by colony forming units.

### ASCs isolated from obese subjects enhance the proliferation of MCF7 cells in vitro

To investigate the effect of the donor’s BMI status and depot site on ASC interaction with breast cancer cells, MCF7 cells or MDA-MB-231 cells were directly co-cultured with ASCs from non-abdominal sources of non-obese subjects (Ob^-^Ab^-^), abdominal source of non-obese subjects (Ob^-^Ab^+^), non-abdominal sources of obese subjects (Ob^+^Ab^-^), or abdominal sources of obese subjects (Ob^+^Ab^+^). Irrespective of the depot site of origin or BMI, ASCs increased the proliferation of MDA-MB-231 cells (*P* <0.01), with no statistically significant difference between the ASC groups (Figure 1A). However, ASCs isolated from obese subjects increased the proliferation of MCF7 cells: 1.5-fold (from 1.1×10^6^ to 1.6×10^6^ MCF7 cells) after co-culture with Ob^+^Ab^-^ ASCs (*P* <0.05) and 2.0-fold (from 1.1×10^6^ cells to 2.2×10^6^ cells) after co-culture with Ob^+^Ab^+^ cells (*P* <0.01; Figure 1A). MCF7 cells co-cultured with ASCs isolated from non-obese subjects failed to increase the cell number.

To determine whether ASCs can influence MCF7 cell growth indirectly, conditioned media was collected from all four ASC groups and added to MCF7 cells. The total number of MCF7 cells was assessed after seven days. MCF7 cells grown in ASC conditioned media from obese subjects increased their proliferation by 1.6-fold (from 0.8×10^6^ to 1.3×10^6^ MCF7 cells after co-culture with Ob^+^Ab^-^ ASCs) and 1.9-fold (from 0.8×10^6^ cells to 1.5×10^6^ cells after co-culture with Ob^+^Ab^+^ cells), respectively (*P* <0.01; Figure 1A); while no statistically significant increase was observed when MCF7 cells were exposed to ASC CM from non-obese donors.

### Donors’ obesity status and depot site of the ASCs influence their effect on the gene expression profile of MCF7 cells

To determine whether the ASCs could induce changes in the gene expression of MCF7 cells, a breast cancer PCR array containing 84 genes known to contribute to breast cancer tumorigenesis and progression was utilized. Of the 84 genes assessed, 13 genes were significantly altered in MCF7 cells by direct co-culture with ASCs, irrespective of the categorical ASC group (*P* <0.05; Table 1). Alterations in the expression of apoptotic, angiogenic, signal transduction, glucocorticoid, metastasis, and xenobiotic transportation genes were observed. These genes included*: BAD, SERPINE1, IL6, CDH13, SNAI2, BIRC5, PGR, ESR1, IGFBP3, NME1, MMP2, MMP9* and *ABCG2* (Table 1). However, the Ob^+^Ab^+^ ASCs altered the expression of an additional 14 unique genes in MCF7 cells (*P* <0.05; Table 1). Alterations in the expression of cell cycle, apoptotic, angiogenesis, metastasis, and adhesion genes were observed. These genes included: *CDKN2A, CCND2, PTEN, SFRP1, PLAU, SLIT2, TWIST1, PTGS2, THBS1, CSF1* and *ADAM23*. The complete comparative analysis of changes in MCF7 gene expression profile after co-culture with ASCs can be found in Additional file 2.

**Table 1:** mRNA expression of MCF7 cells after exposure to ASCs

**Functional gene grouping**	**Gene**	**MCF7 cells gene expression after CC with**			
		**Ob**^**-**^**Ab**^**-**^	**Ob**^**-**^**Ab**^**+**^	**Ob**^**+**^**Ab**^**-**^	**Ob**^**+**^**Ab**^**+**^
Cell cycle	*CDKN2A*	1.41	1.20	2.40	50.33*
	*CCND2*	1.41	1.27	2.40	1.77*
	*PTEN*	1.05	1.04	−1.07	−1.18*
Apoptosis	*GSTP1*	9.79	22.15	17.99	2,475.21*^,#^
	*BAD*	2.21*	2.01*	1.85*	1.33*
	*SFRP1*	1.40	1.23	2.45	56.75^#^
Angiogenesis	*SERPINE1*	15.72*	33.81*	27.27*	4,672.57*^,#^
	*IL6*	3.47*	5.44*	7.01*	197.63*^,#^
	*CDH13*	3.65*	4.02*	4.64*	24.70*^,#^
	*PLAU*	1.99	2.12	3.40	47.07^#^
	*SLIT2*	3.15	2.42	1.05	30.48^#^
Signal transduction					
*Hedgehog*	*SNAI2*	4.47*	11.26*	7.41*	1,592.05*^,#^
*Notch*	*BIRC5*	3.17*	3.36*	3.42*	1.84*^,#^
*Steroid receptor-mediated*	*PGR*	4.15*	5.84*	12.72*	75.41*^,#^
*Steroid receptor-mediated*	*ESR1*	3.97*	4.68*	16.87*	26.85*^,#^
Glucocorticoid	*IGFBP3*	15.47*	32.51*	15.03*	2,746.41*^,#^
	*NME1*	2.86*	3.03*	3.12*	2.02*
Metastasis	*MMP2*	12.22*	24.35*	11.05*	2,320.15*^,#^
	*MMP9*	3.52*	3.59*	4.46*	1.77*^,#^
	*TWIST1*	1.45	1.31	2.51	278.20^#^
	*PTGS2*	1.34	1.15	2.30	81.38^#^
Adhesion	*THBS1*	1.05	1.31	1.16	54.82*
	*CSF1*	1.99	2.12	1.01	42.71*
	*ADAM23*	1.42	1.33	1.27	7.50*
Xenobiotic transport	*ABCG2*	12.56*	12.09*	29.09*^,#^	12.70*

Up-regulated (+) or down-regulated (−) gene expression is shown as fold change relative to MCF7 cells without previous co-culture with ASCs. Values are normalized to MCF7 cells without exposure to ASCs.

*P <0.05; ^#^*P* <0.01.

### Increased expression of CDKN2A, GSTP1, SFRP1, ESR1 and PGR in MCF7 cells after co-culture with ASCs

**Figure 2:**
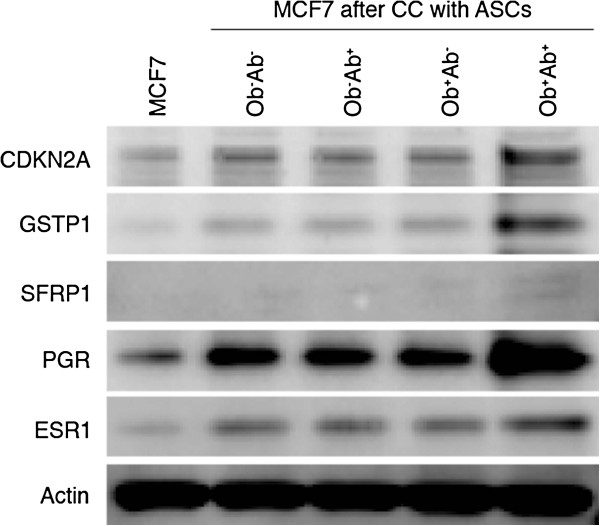
**Changes in the expression of cell cycle regulators and steroid receptors of MCF7 cells.** MCF7 cells were grown alone or in the presence of ASCs (n = 6 donors per group) grouped by the donor’s obesity status and depot site of ASCs for seven days. Cell lysate from sorted GFP^+^ MCF7 cells was collected for Western blot analysis of CDKN2A, GSTP1, SFRP1, ESR1 and PGR. Blots were then stripped and probed for actin as a control.

Western blot analysis was performed to confirm the altered gene expression related to cell cycle control, apoptosis and steroid receptors identified in the MCF7 cells on the PCR array with fold changes greater than five-fold. Cell cycle regulator CDKN2A, apoptotic gene *GSTP1* and *SFRP1*, and the steroid receptors ESR1 and PGR were analyzed. Robust increases in the levels of protein expression for *CDKN2A*, *GSTP1*, *SFRP1*, *ESR1* and *PGR* were observed in MCF7 cells after co-culture with ASCs, irrespective of the ASC group (*P* <0.05; Figure 2). However, co-culture with Ob^**+**^Ab^+^ ASCs induced the most significant fold increase in the expression of *CDKN2A* (from 0.4 to 0.9), *GSTP1* (from 0.3 to 0.9), *SFRP1* (from 0.1 to 0.3), *ESR1* (from 1.1 to 1.7) and *PGR* (from 1.4 to 3.2) (*P* <0.05).

### Estrogen enhances the ASC-induced MCF7 cell proliferation in vitro

**Figure 3:**
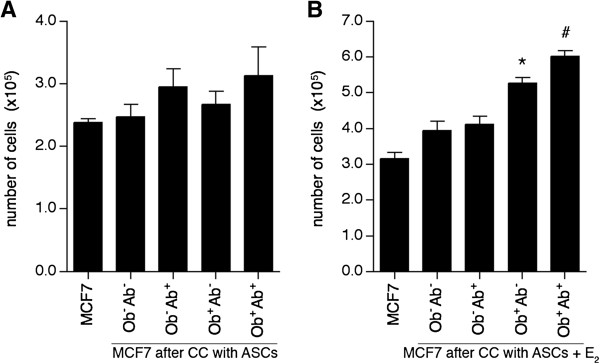
**Estrogen enhances the effect of ASCs on MCF7 proliferation *in vitro*.** Adipose stem cells (ASCs) (n = 6 donors per group) were co-cultured (CC) with MCF7 cells in media supplemented with charcoal dextran stripped fetal bovine serum. **(A)** After seven days, quantification of MCF7 cells was based on the percentage GFP^+^ cells in the population multiplied by the total number of cells in the condition. **(B)** ASCs were grown in 10 nM estrogen (E_2_) replenished every three days, and after seven days, quantification of MCF7 cells was based on the percentage of GFP^+^ cells in the population multiplied by the total number of cells in each condition. Values reported are the mean of three independent experiments, each performed in triplicate. Bars, ± SD. *, *P* <0.05.

Due to the marked increase in ESR1 and PGR expression in MCF7 cells, the effect of estrogen on the ASC-induced MCF7 cell proliferation was assessed. In the absence of estrogen, MCF7 cells demonstrated no significant change in cell proliferation after co-culture with ASCs grown in CCM made with CDS-FBS (*P* >0.05; Figure 3A), indicating that the depletion of estrogen and other growth factors commonly found in FBS eliminated the stimuli that enhanced cellular proliferation. The addition of estrogen increased MCF7 proliferation 1.4-fold (from 2.3×10^5^ cells to 3.1×10^5^ MCF7 cells) after seven days. Estrogen induced proliferation was greatly increased when MCF7 cells were co-cultured with ASCs isolated from obese subjects, increasing the proliferation of MCF7 1.7-fold after co-culture with Ob^+^Ab^-^ ASCs (from 3.1×10^5^ cells to 5.3×10^5^ MCF7 cells) and 1.9-fold after co-culture with Ob^+^Ab^+^ ASCs (from 3.1×10^5^ cells to 6.0×10^5^ MCF7 cells) (*P* <0.05; Figure 3B). While co-culturing MCF7 cells with ASCs isolated from non-obese subjects increased the proliferation of the MCF7 cells, these results were not statistically significant (Figure 3B).

### Tumor volume of MCF7 and ASC xenografts is related to the obesity status and depot source of the ASCs

To assess the role of ASCs in tumorigenesis, MCF7 cells and ASCs were mixed and injected into the mammary fat pad of ovariectomized immunocompromised SCID/beige mice, and tumor volumes were measured regularly over 36 days. In the absence of estrogen, MCF7 xenografts were 140 ± 9.8 mm^3^, while co-mixed xenografts (MCF7 and ASCs) were between 84.0 and 107.5 mm^3^, with no statistically significant difference observed between the four groups of co-mixed xenografts (Additional file 3). These results suggest that in the absence of estrogen, ASCs do not exert any influence on breast cancer tumorigenesis.

**Figure 4:**
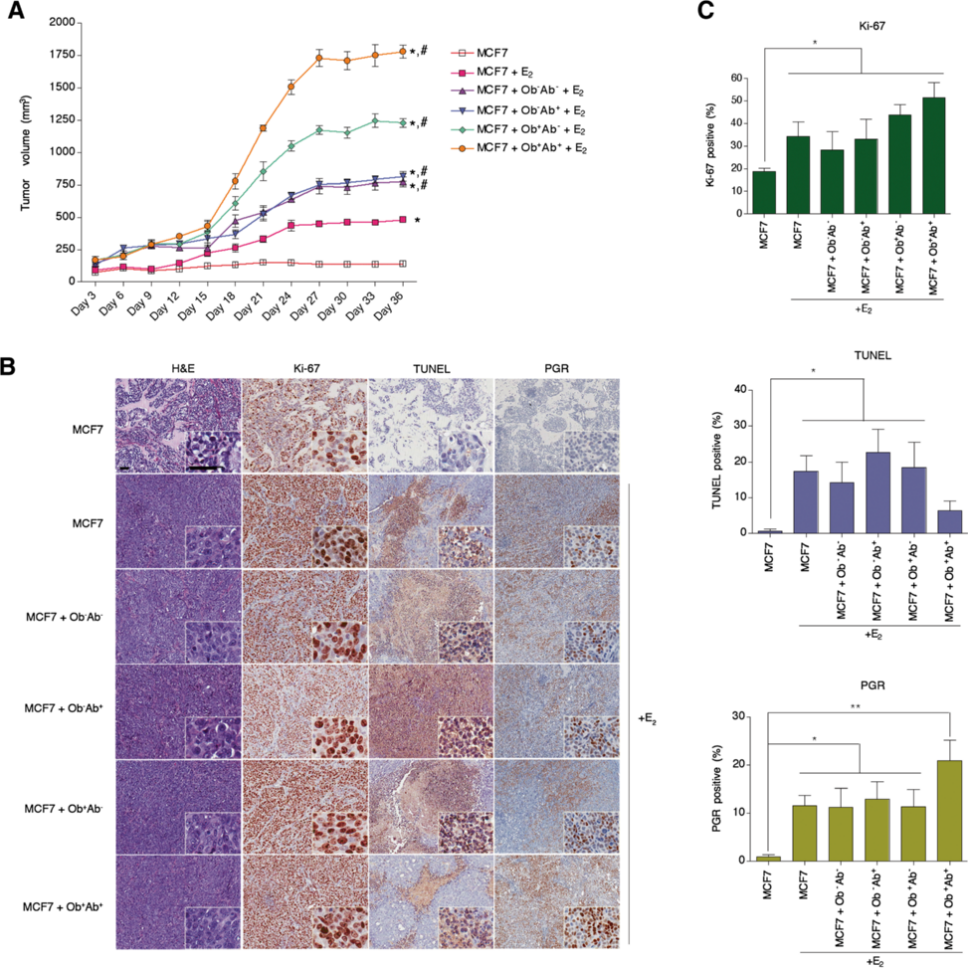
**Donor’s obesity status and depot site of ASCs differentiates its influence on tumorigenicity *in vivo*.** MCF7 cells alone (10^6^) or MCF7 cells and adipose stem cells (ASCs) (n = 6 donors per group) were co-mixed in a 1:1 ratio (10^6^ of each cell type) in a total volume of 50 μl of sterile PBS and mixed with 100 μl of reduced growth factor Matrigel™. Cells were injected into the fifth mammary fat pad of six-week old female ovariectomized SCID/beige mice (*n* = 5 mice per group). Estrogen was delivered subcutaneously in the neck as a 0.72 mg 60-day time-release estrogen pellet. Tumor volume was measured every 3 days for a total of 36 days. **(A)** Tumor volume of tumors formed with MCF7 cells alone or co-mixed cells (MCF7 and ASCs) injected into the mammary fat pad with or without estrogen pellet (E_2_). **(B)** Representative images of H&E, Ki-67, TUNEL and PGR staining of tumor sections from tumor sections taken at 10x and 40x. Scale bars both represent 50 μm. **(C)** Quantification of Ki-67, TUNEL and PGR staining with ImageScope represented as the percentage of positive pixels over total number of pixels per tumor section. Values reported are means of 10 tumor sections. Bars, ± SD. *, *P* <0.05 between MCF7 and other groups. ^#^, *P* <0.01 between MCF7 with estrogen and other groups, **, *P* <0.001 between MCF7 and other groups.

In the presence of estrogen, xenografts formed with MCF7 cells became both easily palpable and visible by Day 36 compared to xenografts in the absence of estrogen (*P* <0.05; Figure 4A). The co-mixed xenografts (MCF7 and ASCs) were significantly larger than the MCF7 only xenografts (*P* <0.01; Figure 4A), suggesting that estrogen may be mediating its effects by activating the ASCs. Tumor volumes of xenografts formed with ASCs isolated from non-obese subjects were similar in size, irrespective of depot site of origin Ob^-^Ab^+^ ASCs (TV = 816.5 ± 36.7 mm^3^) or Ob^-^Ab^-^ ASCs (tumor volume (TV) = 774.4 ± 38.2 mm^3^) (Figure 4A). However, xenografts formed with MCF7 cells and Ob^+^Ab^+^ ASCs (TV = 1,779.6 ± 49.0 mm^3^) were significantly larger in size than xenografts formed with Ob^+^Ab^-^ ASC (TV = 1,230.0 ± 33.5 mm^3^) (*P* <0.05; Figure 4A). In the presence of estrogen, irrespective of MCF7 cell or co-mixed xenografts, increased cellularity was observed, without distinction between the groups (Figure 4B).

In order to assess proliferation and apoptosis, tumor sections were stained with Ki-67 and TUNEL. In the presence of estrogen, co-mixed tumors consisting of ASCs isolated from obese subjects, Ob^+^Ab^-^ ASCs and Ob^+^Ab^+^ ASCs, demonstrated higher proliferation rates than MCF7-only xenografts. However, the co-mixed tumors formed with MCF7 cells and Ob^+^Ab^+^ ASCs demonstrated significantly more expression of Ki-67 (51.5% Ki-67 positive; *P* <0.05; Figure 4B, C). The detection of apoptotic cells demonstrated a lower frequency of apoptosis events in co-mixed tumors formed with MCF7 and Ob^+^Ab^+^ ASCs with 6.5% positive compared to 17.4% positive in MCF7 xenografts (*P* <0.05; Figure 4B, C).

### Enhanced progesterone receptor expression in xenografts formed with Ob^+^Ab^+^ ASCs

Due to the increased tumor volume and decreased levels of apoptosis observed in co-mixed xenografts formed with MCF7 cells and Ob^+^Ab^+^ ASCs in the presence of estrogen, the possibility of estrogen receptor (ER)-mediated signaling was further explored. Tumor sections were stained for progesterone receptor (PGR), as PGR expression is mediated by ER signaling and its increased expression correlates with ER activation. The xenografts formed in the absence of estrogen demonstrated no PGR staining (Figure 4B; Additional file 3). In the presence of estrogen, PGR expression increased in all xenografts and was highest in the MCF7 cell/Ob^+^Ab^+^ ASCs xenografts (20.9% PGR positivity) compared to 11.7% PGR positivity in MCF7 only xenografts (*P* <0.05; Figure 4B, C).

### Gene expression profiles differ between ASCs based on obesity status and depot source

In order to explore quantifiable differences in gene expression between the groups of ASCs, a PCR array with genes known to play a role in obesity was utilized. The results demonstrated that the mRNA expression levels of eight genes were altered between the Ob^-^Ab^+^ ASCs, Ob^+^Ab^-^ ASCs and Ob^+^Ab^+^ ASCs when compared to Ob^-^Ab^-^ ASCs: leptin (LEP), leptin receptor (LEPR), sortilin 1 (SORT1), thyrotropin-releasing hormone (TRH), melanin-concentrating hormone 1 (MCHR1), peroxisome proliferator-activated receptor-gamma (PPAR-gamma), peroxisome proliferator-activated receptor gamma coactivator 1-α (PPARGC1A) and thyroid hormone receptor-β (THRB) (*P* <0.05; Additional file 4; Additional file 5). Furthermore, the Ob^+^Ab^+^ ASCs demonstrated more robust changes in the expression of both LEP (201.5-fold increase) and TRH (50.44-fold decrease; *P* <0.01; Additional file 4; Additional file 5). The full comparative analysis of the gene expression profile of the four groups of ASCs can be found in Additional files 4 and 5.

### Estrogen stimulates leptin expression in ASCs which then enhances MCF7 proliferation

**Figure 5:**
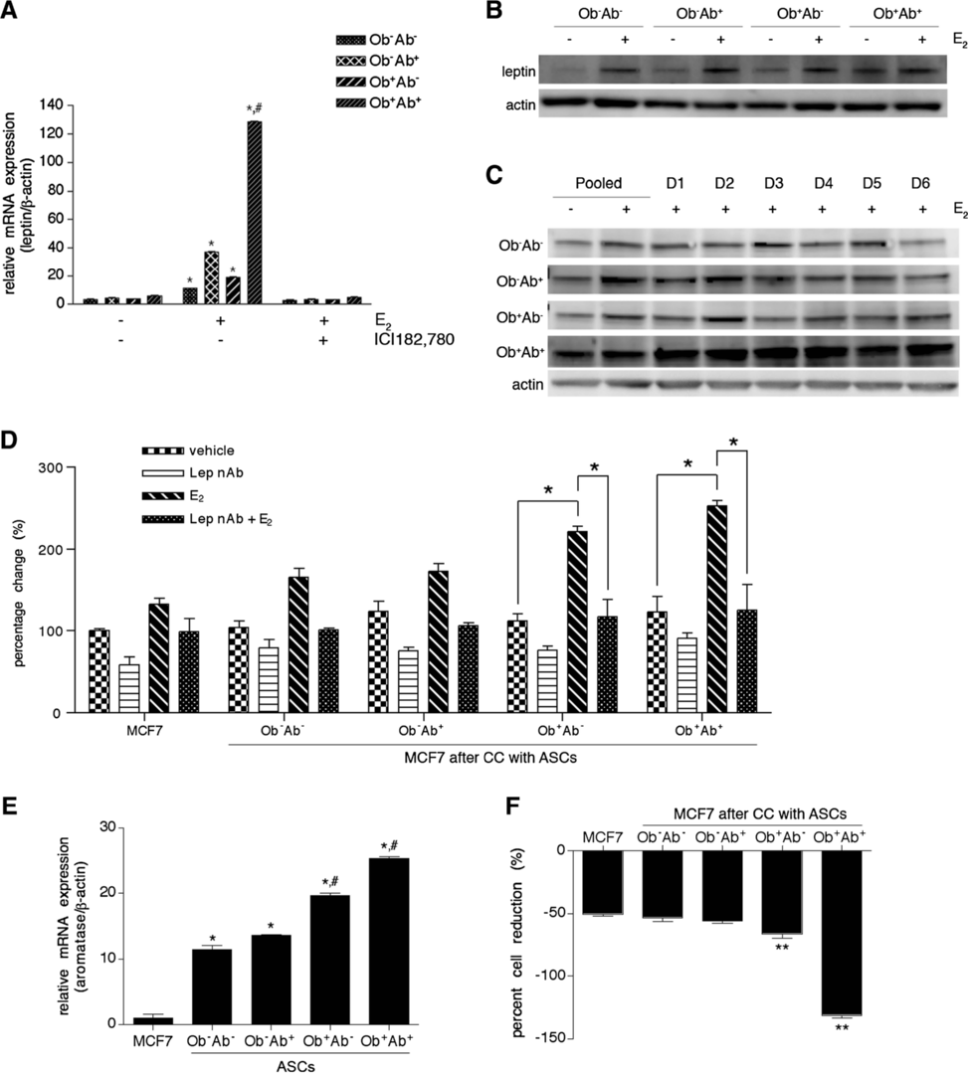
**Estrogen exposure influences leptin expression in ASCs. (A)** Adipose stem cells ASCs (n = 6 donors per group) were cultured in media supplemented with charcoal dextran stripped FBS (CDS-FBS) supplemented with (E_2_) or ICI182,780. Cells were collected at 70% confluency and total RNA was isolated for real-time PCR analysis of leptin expression. **(B)** Cell lysate was collected from n = 6 ASC donors cultured in CDS-FBS with or without E_2_ at 70% confluency and subjected to Western blot analysis with 20 μg of protein and separated by SDS-page under reducing conditions, blotted and probed with antibodies to leptin or actin. **(C)** Cell lysate was collected from six different ASCs donors and either pooled or analyzed as individual donors (D#), cultured in CDS-FBS with or without estrogen. A total of 20 μg of protein was separated by SDS-page under reducing conditions, blotted, and probed with leptin and actin antibodies. **(D)** ASCs (n = 6 donors per group) were co-cultured with MCF7 cells and treated with vehicle (dimethyl sulfoxide, DMSO) neutralizing leptin antibody (Lep nAb), estrogen (E_2_) or neutralizing leptin antibody and estrogen (Lep nAb + E_2_) in complete culture media (CCM) containing CDS-FBS. After seven days, the number of MCF7 cells was calculated based on the percentage of GFP^+^ MCF7 cells by the total number of cells in the population. **(E)** ASCs (n = 6 donors per group) were cultured in CCM and collected at 70% confluency and total RNA was isolated for real-time PCR analysis of aromatase expression. **(F)** ASCs (n = 6 donors per group) were co-cultured with MCF7 cells and treated with letrozole (10 nM) in CCM. After seven days, the number of MCF7 cells was determined by the percentage of GFP^+^ cells multipled by the total number of cells. Percent change was determined by the relative change in the number of MCF7 cells after letrozole treatment compared to their non-treated controls. Values reported are the mean of three independent experiments, each performed in triplicate. Bars, ± SD. *, *P* <0.05; **, *P* <0.01; ^#^, *P* <0.005.

With the increased expression of leptin in Ob^+^Ab^+^ ASCs, the potential ER-mediated response through leptin was explored by exposing ASCs cultured in CDS-FBS to estrogen and/or ICI182,780, a steroidal estrogen antagonist. The removal of endogenous estrogen in the FBS resulted in no statistical alterations of leptin expression between the groups (Figure 5A), with a 3.1-fold increase in Ob^-^Ab^-^ ASCs, 4.4-fold increase in Ob^-^Ab^+^ ASCs, 3.5-fold increase in Ob^+^Ab^-^ ASCs and 5.8-fold increase in Ob^+^Ab^+^ ASCs. However, in the presence of estrogen, the expression of leptin in the ASCs increased from 11.1- (Ob^-^Ab^-^ ASCs), 37.2- (Ob^-^Ab^+^ ASCs), 18.7- (Ob^+^Ab^-^ ASCs), and 128.8-fold (Ob^+^Ab^+^ ASCs) (*P* <0.05; Figure 5A). While all of the groups of ASCs demonstrated an increase in leptin expression, the Ob^+^Ab^+^ ASCs demonstrated the greatest fold increase (10-fold) in leptin expression compared to the other groups (*P* <0.005; Figure 5A). These results suggest robust activation of Ob^+^Ab^+^ ASCs by estrogen. The treatment of the cells with ICI182,780 in the presence estrogen reduced leptin expression in each group to pretreatment levels.

To confirm that the levels of leptin protein increased in the ASCs, cells were cultured in CDS-FBS with or without supplementation of estrogen and probed for leptin expression by Western blot. Leptin levels in each ASC group increased in the presence of estrogen (Figure 5B). In order to prove that these results were not donor dependent, each individual ASC donor was treated with estrogen and probed for leptin expression. While some donor variability was observed between the donors within a group, leptin always increased and the levels of variability were not statistically significant. Moreover, all Ob^+^Ab^+^ ASC donors demonstrated the greatest increase in leptin expression compared to the other ASC donors (*P* <0.01; Figure 5C).

To determine the role of leptin in proliferation of breast cancer cells, MCF7 cells were co-cultured with ASCs in the presence of a neutralizing antibody to leptin. After seven days, in the presence of the neutralizing antibody and estrogen, MCF7 cells did not increase in proliferation, irrespective of the ASC group (Figure 5D). These results indicate that ASCs induce MCF7 proliferation, at least in part, through an estrogen-mediated activation of leptin in ASCs.

### Enhanced ASC aromatase expression and activity increases MCF7 cells

In order to assess local estrogen synthesis, ASCs were grown in CCM and aromatase mRNA expression was assessed. ASCs isolated from obese donors demonstrated enhanced aromatase expression, as Ob^-^Ab^-^, Ob^-^Ab^+^, Ob^+^Ab^-^ and Ob^+^Ab^+^ ASCs demonstrated 11.6-, 13.7-, 19.7- and 25.4-fold increase relative to MCF7 cells, respectively (*P* <0.05; Figure 5E). Furthermore, ASCs isolated from obese donors demonstrated greater aromatase expression compared to non-obese donors (*P* <0.01). Furthermore, delivery of aromatase inhibitors reduced the enhanced proliferation of MCF7 cells due to the co-culture with ASCs. MCF7 cells co-cultured with ASCs from obese subjects demonstrated the most significant reduction, with the most significant decrease in Ob^+^Ab^-^ ASCs (−66.3%) and Ob^+^Ab^+^ ASCs (−131.4%) (*P* <0.01; Figure 5F).

### High leptin levels correlate with poor relapse-free survival in ER^+^/PR^+^ breast cancer

**Figure 6:**
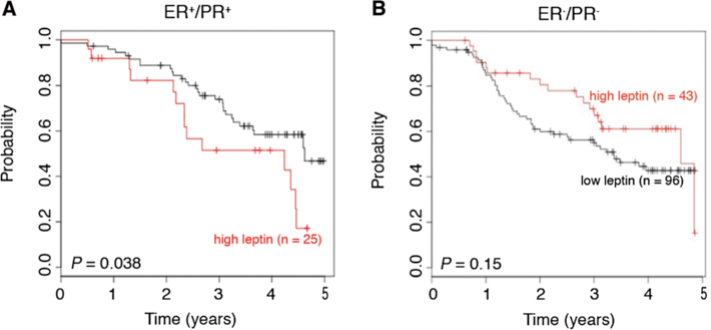
**Levels of leptin expression correlate with decreased survival in ER**^**+**^**/PR**^**+ **^**breast cancers.** Kaplan Meier analysis of the probability of five-year relapse-free survival of women diagnosed with **(A)** ER^+^/PR^+^ or **(B)** ER^-^/PR^-^ breast cancers based on leptin expression.

In order to assess expression of leptin in human breast cancer samples, leptin expression was analyzed using the TCGA cDNA microarray data set of breast cancers deposited in Oncomine. Compared to normal breast tissue (*n* = 73), invasive ductal breast carcinoma (IBC; *n* = 367) demonstrated a 2.0-fold increase in expression (*P* = 0.005). To determine whether leptin expression related to prognosis and the hormone status of the breast cancer, further analysis was conducted with the Kaplan-Meier (KM) Plotter. KM plots demonstrated that women diagnosed with ER^+^/PR^+^ breast cancers whose tumor also expressed high levels of leptin (n = 25) demonstrated poorer prognosis with increased mortality rates as compared to ER^+/^PR^+^ breast cancers with lower levels of leptin (n = 73; *P* = 0.038; Figure 6A). In contrast, analysis of leptin expression in women diagnosed with ER^-^/PR^-^ breast cancer did not demonstrate a correlation between leptin and relapse-free survival outcomes (*P* = 0.15; Figure 6B). Together, this suggests that invasive breast carcinoma overexpresses leptin compared to normal breast tissue and that leptin levels may also be a potential prognostic factor for ER^+^/PR^+^ invasive breast carcinomas.

## Discussion

The results of this study demonstrate that site of origin and BMI alter the biologic properties of subcutaneous human ASCs and their role in cancer tumorigenesis. ASCs were categorized based on depot source and obesity status of the donor subject. ASCs isolated from obese subjects led to the greatest increase in the proliferation and tumorigenicity of MCF7 cells *in vitro* and *in vivo* via an estrogen-activated response mediated through leptin.

Previous studies have explored the endocrine role of adipocytes on breast cancer cell proliferation and metastasis [22]; however, only a few studies have examined the effects of ASCs on breast cancer cell lines. Muehleberg *et al*. demonstrated that ASCs increased the proliferation of a murine breast cancer cell line and enhanced the invasion and metastasis of cancer cells [14]. Utilizing ASCs isolated from the breast and from abdominal adipose tissue, Walter *et al*. demonstrated that the secretion of IL-6 from ASCs enhanced the migration and invasion of breast cancer cells [10]. The results from this study provide further support for the role of ASCs in the tumor microenvironment. The data from this study demonstrated that ASCs effectively alter the gene expression profile of breast cancer cells. More specifically, ASCs isolated from obese subjects enhanced breast cancer cell proliferation and tumor volume and ASCs isolated from the abdomen of obese subjects (BMI >30) further altered the expression of several additional genes. These data provide additional support for the role of obesity, especially abdominal obesity, in affecting breast cancer prognosis. At present, the mechanism(s) by which abdominal obesity induces changes in the ASCs is not known. Rehrer *et al*. demonstrated differences in the subcutaneous adipose tissue isolated from the hip and flank compared to adipose tissue isolated from the abdomen, providing support for differences in adipose tissue based on depot site [23]. More specifically, genes involved in the biochemical metabolism were expressed at higher levels in the abdomen compared to the hip [23]. Additional studies into the microenvironment of the adipose tissue in obese subjects, prior to isolation, would provide substantial insight into the influence of obesity on ASC biology.

Analysis of the gene expression profiles of the four ASC groups revealed significant differences in leptin expression and further determined that the enhanced leptin levels were the direct result of exposure to estrogen. The analysis of the four groups of ASCs, categorized based on depot site and obesity status of the subject, provides support for the potential of ASCs to alter breast cancer cells. More specifically, while leptin expression was increased in ASCs isolated from the abdomen of non-obese subjects and from non-abdominal sources of obese subjects, a more robust increase in leptin expression was observed in ASCs isolated from the abdomen of obese subjects when the cells are exposed to estrogen. Leptin has been shown to play a vital role in the progression of breast cancer through the activation of several signaling cascades. Delivery of leptin to breast cancer cells enhances the proliferation rate through the activation of the STAT3 and ERK1/2 signaling pathway and diminishes apoptosis through a significant reduction in p53 expression and Bax production [24,25]. Several studies suggest that leptin also exhibits estrogen-producing activity by enhancing aromatase expression and enhances the sensitivity of breast cancer cells to estrogen through the up-regulation of estrogen receptor alpha [26,27]. In addition, the inhibition of leptin-signaling results in diminished tumor growth and progression. Animal studies demonstrate that subcutaneous injection of leptin receptor antagonist peptide delayed the development and slowed the growth of breast cancer tumors, suggesting the involvement of leptin in tumor latency and growth [28].

While ASCs isolated from the abdomen of obese subjects demonstrated an increase in leptin expression, the cells failed to elicit an effect on breast cancer cell proliferation or tumor growth. After exposure to estrogen the ASCs increased in leptin expression and breast cancer cell proliferation and tumor growth, suggesting that a threshold of expression must be achieved before leptin can effectively activate breast cancer cell proliferation. These findings indicate that one of the primary mechanism(s) by which ASCs influence breast cancer is via estrogen-mediated pathways. While this study did not focus on the origins of the estrogen, it has been shown that the adipose tissue of obese subjects produce significantly more estrogen through enhanced aromatase activity [29]. As such, enhanced estrogen production in the adipose tissue of obese subjects could potentially stimulate an altered ASC phenotype to secrete an abundance of leptin to alter the gene expression profile of breast cancer cells.

The analysis of the gene expression profiles of breast cancer cells after co-culture with ASCs indicate that the ASCs can activate signaling cascades that enhance proliferation, reduce apoptosis, stimulate angiogenesis and increase metastatic rate of breast cancer cells [10]. The direct co-culture studies revealed the up-regulation of CDKN2A, a cell cycle regulator, and *GSTP1*, a gene responsible for the detoxification of drugs, which have been shown to be up-regulated in multi-drug resistant breast cancer [30]. More specifically, Kars *et al*. demonstrated enhanced *GSTP1* expression and CDKN2A expression among their pacilitaxel and vincristine resistant MCF7 cell lines [30]. These results may suggest that co-culturing ASCs isolated from the abdomen of obese subjects may induce a multi-drug resistant MCF7 phenotype, but additional studies are necessary.

Although the studies described here utilized MCF7 and MDA-MB-231 cell lines, further analysis with additional ER^+^ breast cancer cell lines may provide insight into the full capacity of ASCs to impact different types of breast cancer. Further studies to evaluate the role of additional adipokines in the conditioned media as well as potential contribution of cell-cell interactions are necessary to fully understand the mechanism by which ASCs influence breast cancer tumorigenesis and progression. Nevertheless, our study provides insight into the variability among donors and within donors. While previous studies have demonstrated significant variation in the growth properties, osteogenic capacity and adipogenic capacity of bone marrow-derived stem cells and adipose stem cells, respectively, the cause of the variability between donors and between different aspirates within the same donor have not been previously identified [31,32]. Herein, our study provides insights into the potential for the depot site of origin as well as donor’s obesity status to contribute to donor-to-donor variability and variability observed within the same donor.

The link between high leptin expression and prognosis for breast cancer patients was determined to be significant in ER^+^/PR^+^ breast cancer. The analysis of leptin expression in primary breast cancer samples demonstrated significant differences in normal breast tissue compared to invasive ductal carcinoma [33,34]. It should be noted that these publically available resources do not separate the tumor stroma from the cancer cells, and as such increased leptin expression could correlate with increased expression of leptin in the tumor stroma, where the ASCs are localized. More detailed analysis of primary breast cancer samples is needed to assess the cell type expressing the leptin. However, the assessment of the ASCs from obese and non-obese subjects as well as their depot site suggest that more profound differences could be observed, if obesity status could be taken into account. Since the patient population analyzed in these two resources did not differentiate patients based on obesity status, it would be expected that the results presented here would reveal only a limited scope on the effects of leptin on survival rate. Taken together, the assessment of the effect of ASCs on breast cancer suggests that the tumor microenvironment consisting of these ASCs could dictate the outcomes in obese patients. Moreover, these findings suggest that leptin may be a novel therapeutic target for breast cancer treatment in obese patients.

## Conclusion

This study demonstrates that ASCs are conditioned by their local microenvironment in obese subjects as both the donor’s obesity status and the site of deposition contribute to the stimulatory effects of ASCs on breast cancer cell growth. Furthermore, the positive effects of estrogen on leptin expression in ASCs suggest a potential mechanism by which these ASCs are elicited to influence breast cancer cell proliferation and suggest a new avenue to be explored for breast cancer treatment in obese patients.

## Abbreviations

αMEM: Alpha-minimum essential medium; Ab-: Non-abdominal source of adipose tissue; Ab+: Abdominal source of adipose tissue; ABCG2: ATP-binding cassette sub-family G, member 2; ADAM23: ADAM metallopeptidase domain 23; ASCs: Adipose stem cells; BAD: BCL2-associated agonist of cell death; BIRC5: Baculoviral IAP repeat containing 5; BMI: Body mass index; BSA: Bovine serum albumin; CCM: Complete culture media; CCND2: Cyclin D2; CDH13: Cadherin 13; CDKN2A: Cyclin dependent kinase inhibitor 2A; CDS-FBS: Charcoal dextran stripped-fetal bovine serum; CFU: Colony forming units; CSF1: Colony stimulating factor 1; DMEM: Dulbecco’s modified eagle’s Medium; E2: Estrogen; ESR1: Estrogen receptor-alpha; FBS: Fetal bovine serum; FFPE: Formalin-fixed paraffin-embedded; GSTP1: Glutathione S-transferase P; HRP: Horseradish peroxidase; ICI182,780: Fulvestrant; IDC: Invasive ductal carcinoma; IGFBP3: Insulin-like growth factor binding protein 3; IL-6: Interleukin-6; LEP: Leptin; LEPR: Leptin receptor; MCHR1: Melanin-concentrating hormone 1; MMP2: Matrix metallopeptidase 2; MMP9: Matrix metallopeptidase 9; NME1: NME/NM23 nucleoside diphosphate kinase 1; Ob-: Lean donor; Ob+: Obese donor; P/S: Penicillin/streptomycin; PBS: Phosphate buffered saline; PBST: Phosphate-buffered solution with 0.01% TWEEN-20; PGR: Progesterone receptor; PLAU: Plasminogen activator; PPAR-gamma: Peroxisome proliferator-activated receptor gamma; PPARGC1A: Peroxisome proliferator-activated receptor gamma coactivator 1-alpha; PTEN: Phosphatase and tensin homolog; PTGS2: Prostaglandin-endoperoxide synthase 2; SD: Standard deviation; SERPINE1: Plasminogen activator inhibitor type 1; SFRP1: Secreted frizzled-related protein 1; SLIT2: Slit homolog 2; SNAI2: Snail homolog 2; SORT1: Sortilin1; TCGA: The Cancer Genome Atlas; THBS1: Thrombospondin 1; THRB: Thyroid hormone receptor-beta; TRH: Thyrotropin-releasing hormone; TWIST1: Twist basic helix-loop-helix transcription factor 1.

## Competing interests

The authors declare no competing financial interests.

## Authors’ contributions

ALS took part in the conception and design of the study, the collection and assembly of data, data analysis and interpretation, and manuscript writing. TAS, XZ, ZS and SZ collected data. LVR and JAS took part in the conception and design of the study and the collection of data. JMG obtained and processed samples from lipoasiprates. MEB provided breast cancer cell lines. JMG, MEB and BAB took part in the conception and design of the study, data interpretation, manuscript writing and financial support. All authors approved the final manuscript.

## Supplementary Material

Additional file 1 **Characterization of ASCs isolated from donors based on obesity status and deposit site. (A)** ASCs (n = 24) in each of the four groups were stained with antibodies against the indicated antigens and analyzed by flow cytometry. Represented cell surface marker profiles for each group are shown. Histograms are shown as colored lines and the respective isotype controls are in gray. **(B)** CFUs were seeded at low density and incubated in CCM for 14 days. Cells were fixed and stained with crystal violet. Images were captured with a digital camera. Representative images for each group are shown. **(C)** ASCs were grown until 70% confluent in CCM and then switched to differentiation media. After 21 days, cells were fixed and stained with Alizarin Red for osteogenesis and Oil Red O for adipogenesis. Representative images for each group are shown. Original magnification for osteogenesis is 4× and for adipogenesis is 10× for all panels. Scale bars represent 100 μm. Click here for file

Additional file 2 **Cluster diagram of relative gene expression of MCF7 cells co-cultured with ASCs characterized by obesity status and depot site of origin.** Expression is relative to MCF7 cells without exposure to ASCs. Click here for file

Additional file 3 **Tumorigenesis of MCF7 cells when co-mixed with the 4 categorical ASC groups in the absence of estrogen. (A)** Tumor volume of MCF7 cells alone or co-mixed cells injected into the mammary fat pad in the absence of estrogen. **(B)** Representative images of immunohistochemistry staining for human Ki-67, TUNEL, and PGR staining in tumor sections. **(C)** Quantification of Ki-67, TUNEL and PGR staining with ImageScope represented as the percentage of positive pixels over total number of pixels per tumor section. All images were acquired at 10× and 40×. Scale bar represents 50 μm. Bars, ± SD. *, *P* <0.05. Click here for file

Additional file 4 **Cluster diagram of relative gene expression of ASCs characterized by obesity status and depot site of origin.** Expression is relative to Ob^-^Ab^-^ ASCs. Click here for file

Additional file 5 **Fold change in mRNA expression of ASCs based on obesity status and depot site of origin.** Values are normalized to Ob^-^Ab^-^ ASCs. *, *P* <0.05; #, *P* <0.01. Click here for file
