# Supplementary figures and images for: Obesity associated alterations in the biology of adipose stem cells mediate enhanced tumorigenesis by estrogen dependent pathways
Source: Breast Cancer Res. 2013 Oct 31;15(5):R102. doi: 10.1186/bcr3569 (PMC3978929; doi:10.1186/bcr3569)

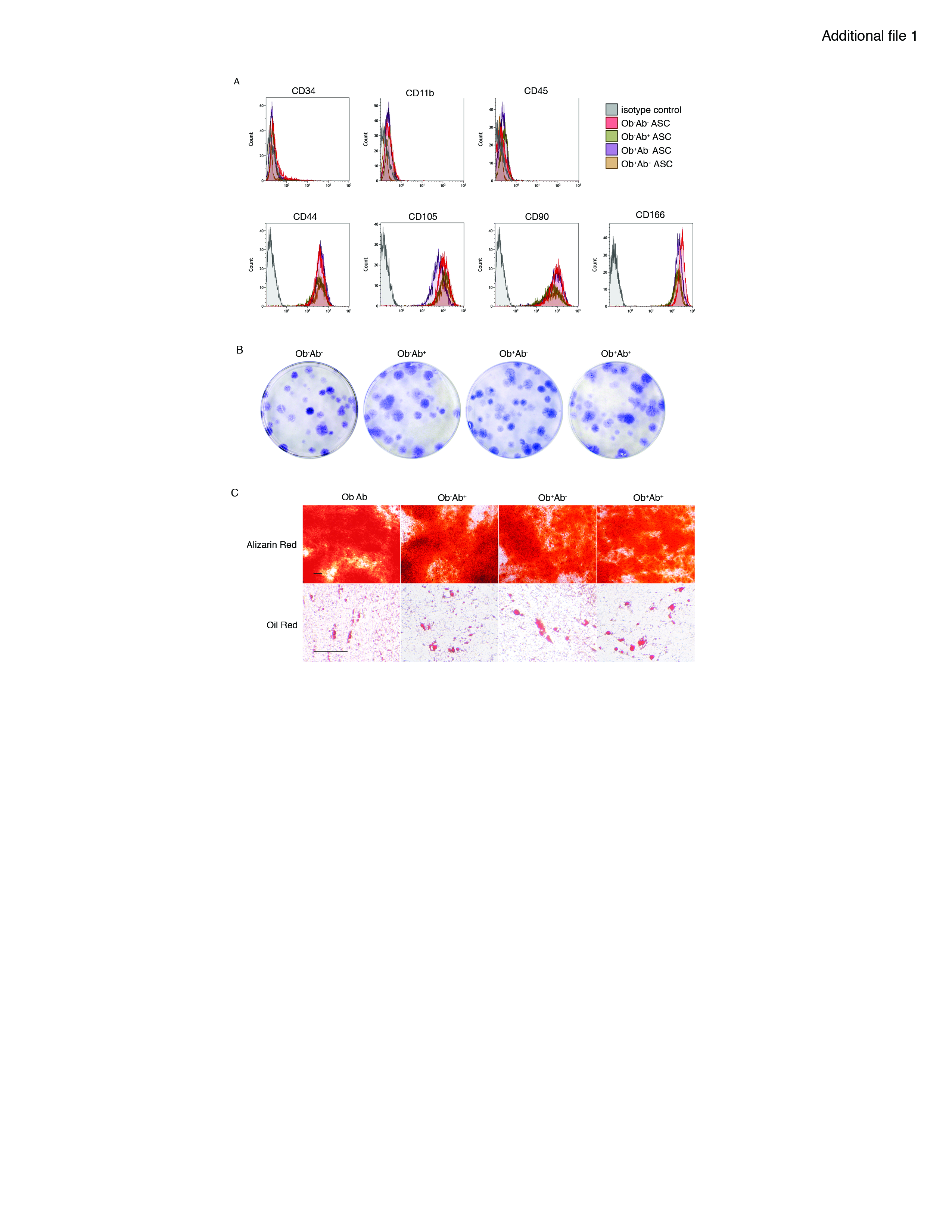

Supplement: Additional file 1 — Characterization of ASCs isolated from donors based on obesity status and deposit site. (A) ASCs (n = 24) in each of the four groups were stained with antibodies against the indicated antigens and analyzed by flow cytometry. Represented cell surface marker profiles for each group are shown. Histograms are shown as colored lines and the respective isotype controls are in gray. (B) CFUs were seeded at low density and incubated in CCM for 14 days. Cells were fixed and stained with crystal violet. Images were captured with a digital camera. Representative images for each group are shown. (C) ASCs were grown until 70% confluent in CCM and then switched to differentiation media. After 21 days, cells were fixed and stained with Alizarin Red for osteogenesis and Oil Red O for adipogenesis. Representative images for each group are shown. Original magnification for osteogenesis is 4× and for adipogenesis is 10× for all panels. Scale bars represent 100 μm. [file bcr3569-S1.tiff]

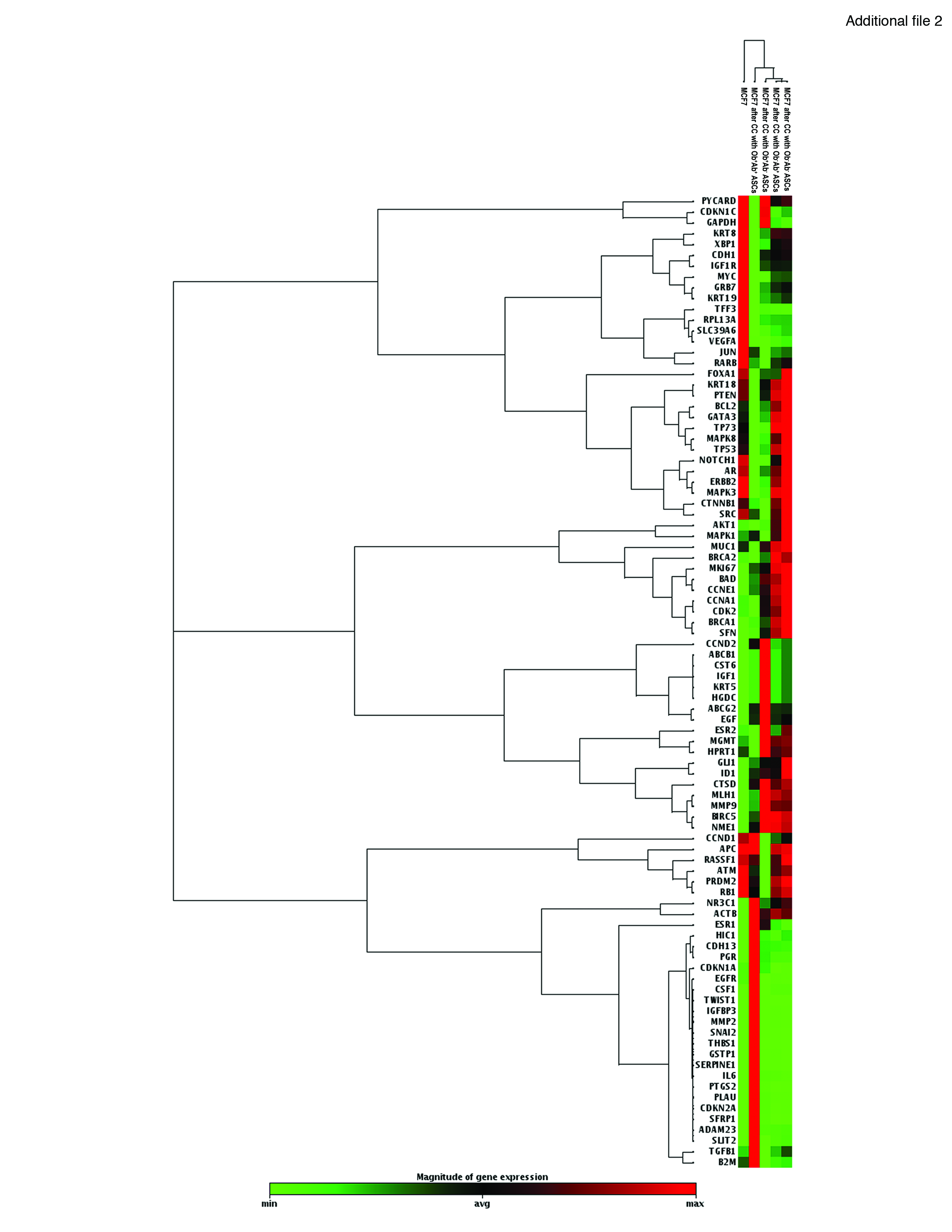

Supplement: Additional file 2 — Cluster diagram of relative gene expression of MCF7 cells co-cultured with ASCs characterized by obesity status and depot site of origin. Expression is relative to MCF7 cells without exposure to ASCs. [file bcr3569-S2.tiff]

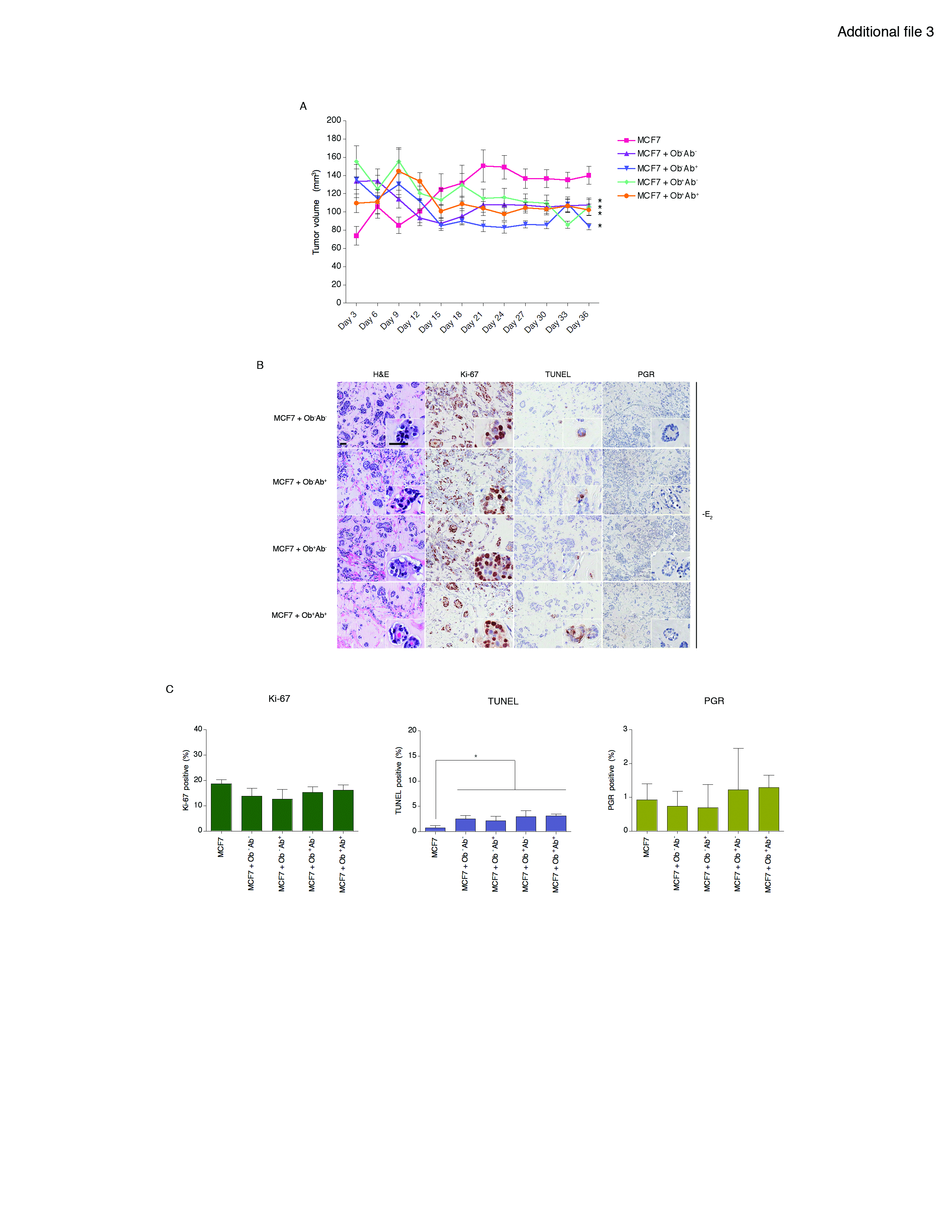

Supplement: Additional file 3 — Tumorigenesis of MCF7 cells when co-mixed with the 4 categorical ASC groups in the absence of estrogen. (A) Tumor volume of MCF7 cells alone or co-mixed cells injected into the mammary fat pad in the absence of estrogen. (B) Representative images of immunohistochemistry staining for human Ki-67, TUNEL, and PGR staining in tumor sections. (C) Quantification of Ki-67, TUNEL and PGR staining with ImageScope represented as the percentage of positive pixels over total number of pixels per tumor section. All images were acquired at 10× and 40×. Scale bar represents 50 μm. Bars, ± SD. *, P <0.05. [file bcr3569-S3.tiff]

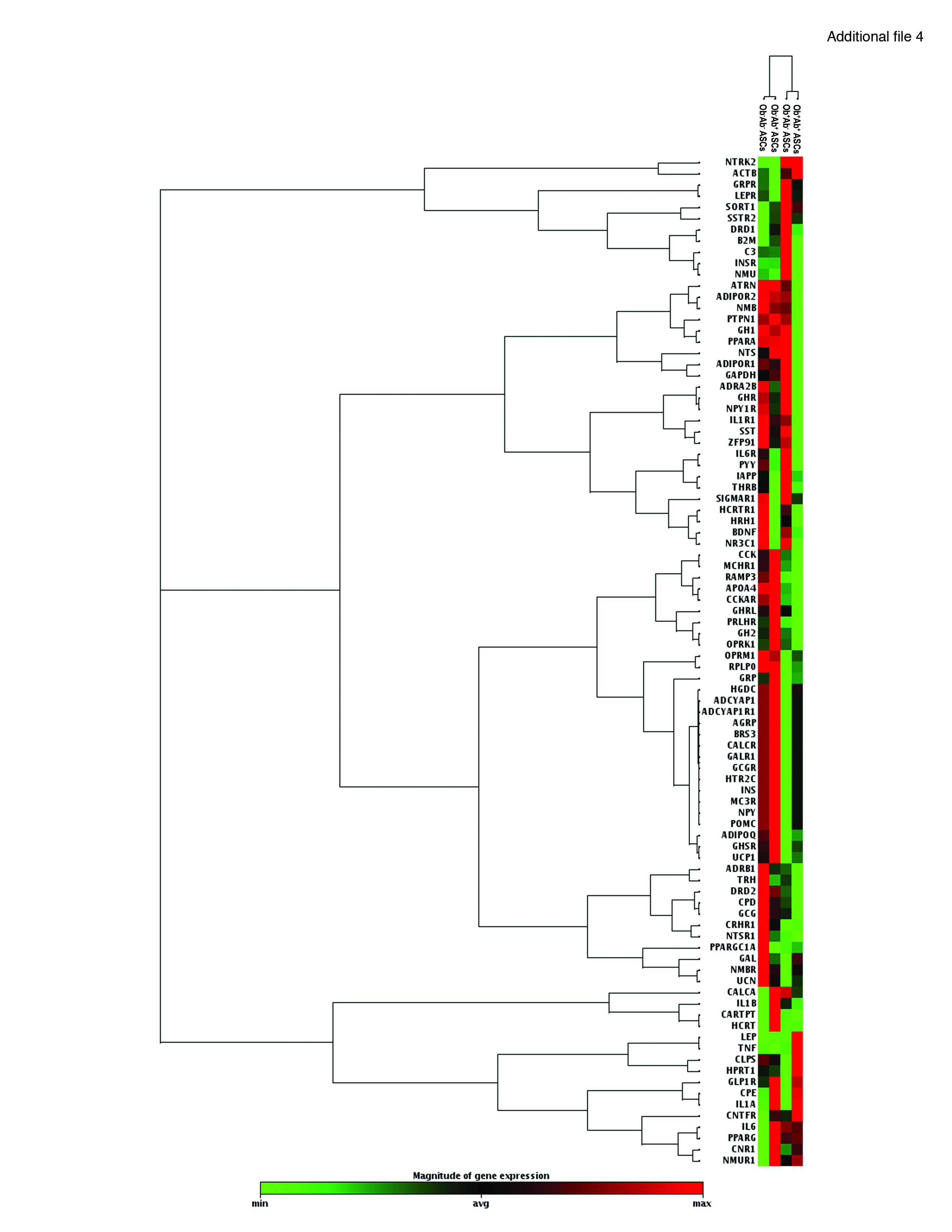

Supplement: Additional file 4 — Cluster diagram of relative gene expression of ASCs characterized by obesity status and depot site of origin. Expression is relative to Ob-Ab- ASCs. [file bcr3569-S4.tiff]
