# Supplementary material for: Obesity associated alterations in the biology of adipose stem cells mediate enhanced tumorigenesis by estrogen dependent pathways
Source: Breast Cancer Res. 2013 Oct 31;15(5):R102. doi: 10.1186/bcr3569 (PMC3978929; doi:10.1186/bcr3569)
Supplement: Additional file 5 — Fold change in mRNA expression of ASCs based on obesity status and depot site of origin. Values are normalized to Ob-Ab- ASCs. *, P <0.05; #, P <0.01. [file bcr3569-S5.doc]

| Functional Grouping | Hormone and Receptor Grouping | Gene | Ob^-^Ab^+^ | Ob^+^Ab^-^ | Ob^+^Ab^+^ |
| --- | --- | --- | --- | --- | --- |
| Anorectic | Adipocyte-derived | LEP | 9.47* | 7.72* | 201.47*^,#^ |
|  |  | LEPR | -1.23* | 1.35* | 3.34* |
|  | Neuro | SORT1 | 1.49* | 2.37* | 1.92* |
|  |  | TRH | -4.25* | -2.52* | -50.44*^,#^ |
| Orexigenic | Neuro | MCHR1 | 1.30* | -1.50* | -2.20* |
| Energy Expenditure | Adipocyte-derived | PPARG | 1.55* | 1.37* | 1.40* |
|  |  | PPARGC1A | -2.48* | -2.19* | -2.16* |
|  | CNS-derived | THRB | -2.21* | 1.49* | -1.84* |

^a^ Data is shown as fold change relative to Ob^-^Ab^-^ ASCs.

^b^ * *P* < 0.05; ^#^ *P* < 0.01
